# Mineral Composition and Bioaccessibility in Rocket and Purslane after Zn Biofortification Process

**DOI:** 10.3390/foods11030484

**Published:** 2022-02-07

**Authors:** Massimiliano D’Imperio, Francesco Fabiano Montesano, Francesco Serio, Elisa Santovito, Angelo Parente

**Affiliations:** Institute of Sciences of Food Production, CNR—National Research Council of Italy, Via Amendola 122/D, 70126 Bari, Italy; massimiliano.dimperio@ispa.cnr.it (M.D.); francesco.montesano@ispa.cnr.it (F.F.M.); francesco.serio@ispa.cnr.it (F.S.); elisa.santovito@ispa.cnr.it (E.S.)

**Keywords:** in vitro digestion process, floating system, baby leaf, hazard quotient, RDA

## Abstract

Zinc (Zn) is an essential key nutrient in different biochemical and physiological processes. The nutritional deficit of this mineral element is estimated to affect the health of over 3 billion people worldwide. Several strategies are available to reduce the negative impact of mineral malnutrition; among them, biofortification is the practice of deliberately increasing the nutrients and healthy compounds in the edible parts of vegetables. This study aims to evaluate Zn bioaccessibility in biofortified and non-biofortified rocket and purslane using an in vitro gastrointestinal digestion process and measure the concentration of other mineral elements (Al, B, Ca, Fe, K, Mg, Mn, and Sr) released during the digestion process from rocket and purslane biofortified with Zn. The bioaccessible Zn in biofortified rocket and purslane ranged from 7.43 to 16.91 mg/kg, respectively. In addition, the daily intake, the RDA coverage (%), and the hazard quotient (HQ) for the intake of Zn (resulting from the consumption of 100 g of rocket and purslane) were calculated. The calculated HQ highlights the safety of these baby leaf vegetables. The study confirms that it is possible to obtain Zn-biofortified rocket and purslane with high Zn bioaccessibility by adopting an appropriate mineral plant nutrition solution enriched in Zn.

## 1. Introduction

Zinc (Zn) is an essential key nutrient for several biochemical activities, such as human growth and development, immune system functions, and gene regulation. After iron, Zn is the second most abundant metal ion in organisms [1,2].

The Zn content in vegetables is related to various factors, such as species, genotype, type of edible portion (seed, leaf, fruit, or roots), phenological stage (microgreens, baby leaf, or mature vegetables), production method, and type of soil [3,4,5,6]. The recommended dietary allowance (RDA) of Zn for adults is 11 mg/day for men and 8 mg/day for women [7]. However, in some physiological conditions (such as pregnancy and lactation), chronic diseases (such as liver cirrhosis), diet (vegans/vegetarians), and in the elderly, it is necessary to increase the Zn intake with nutrition [7]. In humans, Zn deficiency is mostly associated with poor nutrition and poor dietary variegation and is aggravated by its poor availability in soils [5].

Zn deficiency is estimated to affect more than 3 billion of the world’s population, with the vast majority occurring in underdeveloped countries [8,9].

The human and economic cost of Zn malnutrition is noteworthy, considering that about 17% of the global population suffers from this condition in developed and underdeveloped countries. More than 100,000 deaths per year in children under the age of 5 with various pathologies are attributable to the Zn deficiency [1,2,3,4,5,6,7,8,9]. Consequently, a series of international actions have been undertaken to improve the nutritional status of the population exposed to Zn malnutrition through the use of different approaches [10,11,12,13]. Among these, biofortification is the practice of deliberately increasing nutrients and healthy compounds and/or decreasing antinutritional factors (such as phytic and oxalate acids) in plant-based foods (cereal, vegetables, and fruit) [14,15]. Biofortified crops can be obtained through various strategies, such as genetic engineering, plant breeding, and agronomic practices [14,15].

Agronomic biofortification is generally used to increase the content of mineral nutrients (iodine, silicon, calcium, iron, zinc, magnesium, selenium, and copper) in the edible parts of various leafy vegetables and fruits, such as mizuna, tatsoi, chicory, basil, purslane, lettuce, tomato, Swiss chard, rocket, potatoes, green beans, and others [16]. This approach can be applied in different cultivation conditions, such as open field, greenhouse, and indoors; in the latter cases, also using soilless cultivation systems. Indeed, several studies have reported that the efficiency of biofortification, especially in greenhouses and indoor cultivation, can be maximized by specific management of the growing conditions [17,18,19]. The concentration of the nutrient solutions (NS) is an important characteristic for the quality of vegetables production [18]; therefore, changes in the composition of the NS can have a considerable impact on the nutritional quality of products, in particular, on the content of mineral elements [17,19] and bioactive organic compounds [20]. Furthermore, the choice of the plant species for biofortification represents an important aspect of the mineral biofortification process due to the effect of the phylogenetic heritage that inevitably affects plants’ ability to accumulate essential mineral elements [21]. As an example, among leafy vegetables, purslane is considered a “new crop” for ready-to-eat products [22] and is characterized by a high oxalate content (2000 mg/kg of fresh weight). Rocket, on the other hand, is one of the most popular species grown in Mediterranean areas as a “ready-to-eat fresh-cut salads” product and is generally considered oxalate-free [23].

A crucial step after the biofortification process is the assessment of the bioaccessibility of the target nutrient. Ideally, in a successful biofortification protocol, the increase of a target nutrient in the edible parts parallels an increase in its bioaccessibility. The amount of nutrient that is released from the plant matrix during the gastrointestinal digestion process and its evaluation are independent of the approach and the method used to produce the biofortified crop. Furthermore, not all parts of a nutrient in the edible parts of biofortified vegetables can perform a biological activity. The release of nutrients in the intestinal tract (during the gastrointestinal digestion process) depends on different factors, such as species and type, and is subject to various influences, for example the concentration of nutrients, the activity of antinutritional compounds, texture, food processing, and the interaction of some nutrients with others [24,25,26]. During the gastrointestinal digestion process, the interaction of different mineral elements with similar electronic configurations (Zn^2+^, Ca^2+^, Fe^2+^, Mg^2+^, Mn^2+^, and Sr^2+^) can often lead to changes in the bioaccessibility and bioavailability of mineral nutrients [27,28]. Several methods are available to assess bioaccessibility using the in vitro digestion protocol. In these methods, the chemical, physical, and dynamic conditions of the gastrointestinal tract (mouth, stomach, and gut) are artificially reproduced in vitro [29].

Overall, the assessment of bioaccessibility provides information on the number of nutrients released from the food matrix, on nutrient–nutrient and nutrient–antinutrient interactions, on biochemical transformations, on chemical degradations, and on the effect of the matrix [30,31]. Furthermore, the assessment of bioaccessibility represents the starting point for the estimation of the beneficial effects of biofortified products on human health and can be used as a method to improve the food design process.

With all the above taken into account, the objectives of this study were: (i) to evaluate the overall mineral profile of rocket and purslane subjected to a process of Zn biofortification; (ii) to assess the quantity of mineral elements released by biofortified vegetables during the digestion process (bioaccessible fraction); and (iii) to calculate the RDA coverage and the hazard quotient (HQ) in relation to Zn bioaccessibility.

Two baby leaf vegetables (rocket and purslane) were produced and biofortified with Zn, the consumption of which allows an increase of zinc intake in the human diet without causing harm to the consumer. A workflow was proposed that was based on the evaluation of the efficiency of the biofortification process from a nutritional point of view, taking into account the overall bioaccessibility of the mineral nutrients.

## 2. Materials and Methods

### 2.1. Production of Zn-Biofortified Purslane and Rocket

Zn-biofortified rocket and purslane were produced in the experimental greenhouse “La Noria” located in Mola di Bari (BA), southern Italy (41°03′ N, 17°04′ E; 24 m a.s.l.) by using the floating hydroponic system. Rocket and purslane were grown in a complete NS with macro- and micro-nutrients [32]. Zn levels in the NS were 0.13 and 5.2 mg/L for growing non-biofortified and biofortified plants, respectively. The plants were harvested at the commercial stage of “baby leaf” (24 January 2020 and 30 July 2020, respectively, for rocket and purslane), as defined by Di Gioia et al. [33].

### 2.2. Mineral Profile of Rocket and Purslane

Al, B, Ca, Fe, K, Mg, Mn, Na, Sr, and Zn content was measured in dry samples by inductively coupled plasma optical emission spectrometry (ICP-OES) after mineralization of the dry samples with an acid microwave-assisted digestion system (MARS 6, CEM Corporation, Matthews, North Carolina) performed as reported by D’Imperio et al. [34]. To confirm the accuracy of the measurements, certified reference vegetable material (CRM, NIST tomato leaf 1535a) was analyzed using the same procedure as the rocket and purslane samples.

### 2.3. In Vitro Gastrointestinal Digestion Process

The assessment of mineral bioaccessibility (Al, B, Ca, Fe, K, Mg, Mn, Sr, and Zn) from plant samples (biofortified and not) during the digestion process was performed as reported by Ferruzzi et al. [35]. After the digestion process, samples were centrifuged at 10,000× *g* for 1 h at 4 °C to separate the aqueous intestinal digesta, called ‘bioaccessible fraction’ (BF), from the residual solids. The BFs were collected, filtered (0.2 μm PTFE filter), and dried at 50 °C for 48 h before the minerals content was measured. For the CRM sample only, the residual solids were washed with Milli-Q H_2_O (18 MΩ/cm) and dried (50 °C for 48 h) until use. To evaluate the accuracy of the measurement, CRM (NIST tomato leaf 1535a) was analyzed using the same procedure adopted for the rocket and purslane samples.

### 2.4. Analysis of Mineral Content in Digested Sample

After the digestion process, the BF and the residual solid were mineralized with HNO_3_ 65% using the same protocol used for rocket and purslane (see Section 2.2). Blank correction was performed in all analyses. The protocol applied did not allow the estimation of Na bioaccessibility, because the blank correction was not performed for this mineral element. The amount of Na released from the food matrix during the digestion process was lower than the amount of Na in the blank sample (3.81 g/L). This is related to the reagents used, as also reported by another study [36]. The bioaccessibility fraction percentage (BF_%_), defined as the percentage of nutrient(s) released from the digested matrix in the gastrointestinal digestion process, was calculated as BF_%_ = (total nutrient released during digestion/total nutrient in food) × 100.

### 2.5. Percentage of Recommended Daily Allowance and Hazard Quotient for Zn Intake

The recommended daily allowance of Zn (RDA-Zn) is equal to 11 and 8 mg, respectively, for male and female adults [7]. The daily intake of Zn and the percentage of coverage of RDA for Zn (% RDA-Zn) were calculated in relation to the quantity of Zn released from the vegetables during the gastrointestinal digestion process. Risk assessment was also performed by using HQ, considered as the risk to consumer health resulting from the consumption of Zn-biofortified, fresh baby leaf vegetables, based on a 70 kg adult. The HQ is the ratio of the potential exposure to an organic and/or inorganic substance and the level at which no negative effects are expected. HQ allows the estimation of the potential negative effects on health related to chronic consumption of food (in our case, biofortified rocket and purslane). A HQ lower or equal to 1 indicates that adverse effects are unlikely to occur, and, thus, the product can be considered to have negligible hazard. For a HQ greater than 1, the potential for adverse effects increases [37]. The contribution of Zn from other nutritional sources was not examined. The HQ was calculated according to the protocol described by the Environmental Protection Agency [37], using the following equation: HQ = ADD/RFD, where ADD is the average daily dose of Zn (mg of Zn/kg body weight/day), and RFD is the recommended dietary tolerable upper intake level of Zn (mg of Zn/kg body weight/day). The I RFD value for a 70 kg adult is 3 × 10^−1^ mg Zn/kg/day [38]. The ADD for 100 g portions of rocket or purslane was computed as follows: ADD = (MI × CF × DI)/BW. MI is the Zn concentration released during the gastrointestinal digestion process after the consumption of the two vegetables (mg/kg DW); CF is the fresh-to-DW conversion factor for vegetable samples (calculated as the ratio of FW to DW; rocket: 0.093 on average; purslane: 0.054 on average); DI is the daily intake of baby leaf vegetables (kg, taken as 100 g); BW is the body weight (kg) of humans, assumed as 70 kg.

### 2.6. Statistical Analysis

The effects of the biofortification process were evaluated using one-way analysis of variance (ANOVA) followed by means separation with Fisher’s protected least significant difference (LSD) at *p* ≤ 0.05. In the bioaccessibility parameter analysis, the effects of treatments and species were estimated using a two-way analysis of variance (ANOVA) followed by means separation with Fisher’s protected least significant difference (LSD) at *p* ≤ 0.05. The software Statistica 10.0 (StatSoft, Tulsa, OK, USA) was used.

## 3. Results and Discussion

### 3.1. Mineral Analysis

Analysis of the accuracy of the analytical measurements of macro and trace elements in the edible parts and in digested samples, from biofortified and non-biofortified baby leaf vegetables, was performed. The mineral elements Al, B, Ca, Fe, K, Mg, Mn, Sr, and Zn were detected and measured. The limits of detection (LOD) and the limit of quantification (LOQ) of the methods were calculated as suggested by D’Imperio et al. [34]. Tomato leaves (NIST-1535a) were used as CRM to evaluate the accuracy of the measurements in the plants and in the digested samples, as reported in Table 1 and Table 2. The recovery of mineral elements in the vegetable samples ranged from 90 to 107%. After the in vitro digestion of the CRM, some trace elements, such as Al, Fe, and K, showed the lowest recovery values (%), whereas B, Ca, Mg, Mn, Sr, and Zn showed higher recovery values, as reported in Table 2.

### 3.2. Mineral Profile of Biofortified and Non-Biofortified Rocket and Purslane

The biofortification process aims to improve the nutritional value of crops without altering the performance of the crops. In both species, the agronomic protocol applied in this study did not cause any toxic effect in the vegetables nor alteration of the crop performances (data not shown).

**Figure 1:**
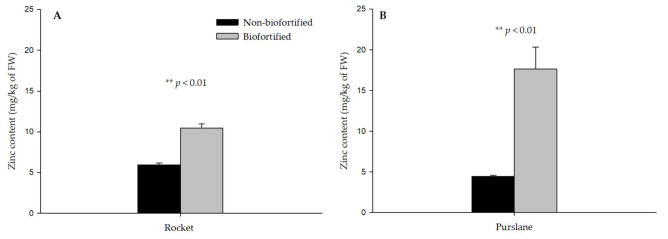
Zinc content in non-biofortified and biofortified rocket (**A**) and purslane (**B**), harvested at the phenological stage of “baby leaf vegetables”. Results are reported as mean ± standard error of treatment (*n* = 3). Means separation within columns by LSD (α = 0.05). Significance: ** *p* < 0.01. Non-biofortified (0.13 mg/L of Zn in nutrient solution), Biofortified (5.2 mg/L of Zn in nutrient solution).

Using 5.2 mg/L of Zn in the NS, the tissue content of Zn in the edible parts of rocket and purslane increased, respectively, by 1.76 and 3.97-fold compared with the non-biofortified counterpart (0.13 mg/L of Zn), as reported in Figure 1. According to our results, the level of Zn used in the biofortification treatment favored its absorption. In fact, zinc is absorbed by plants from the soil as an ionic element or bound to an organic acid and transported through the xylem to the aerial parts (shoots and leaves) [39]. Similar increases in Zn content were found in lettuce [40], cabbage [41], soybean sprouts [42], and in three different types of microgreens that were produced in soilless systems using different levels of Zn in the NS [43].

The content of Al, B, Ca, Fe, K, Mg, Mn, and Sr measured in rocket and purslane did not reveal significant differences imputable to biofortification (Table 3). The overall mean contents (mg/kg of FW) were 3.32 (Al), 2.69 (B), 3364 (Ca), 6.68 (Fe), 7371 (K), 520 (Mg), 2.24 (Mn), and 6.17 (Sr) in rocket and 0.89 (Al), 2.36 (B), 940 (Ca), 4.07 (Fe), 4279 (K), 856 (Mg), 8.55 (Mn), and 2.77 (Sr) in purslane. In our study, no antagonistic effects were found between Zn and other mineral elements, such as K, Ca, and Fe, although this kind of antagonism has been reported in other studies and is related to the fact that these mineral elements share the same transporters on the plasma membrane [44]. However, our result could be related to the low Zn level used in this study (5.2 mg/L of Zn in NS). Di Gioia et al. [43] reported antagonistic effects between Zn and the other mineral elements using higher levels of Zn in the NS (10 and 20 mg/L) than the level used in this study.

### 3.3. Mineral Bioaccessibility in Rocket and Purslane after the Biofortification Process

**Figure 2:**
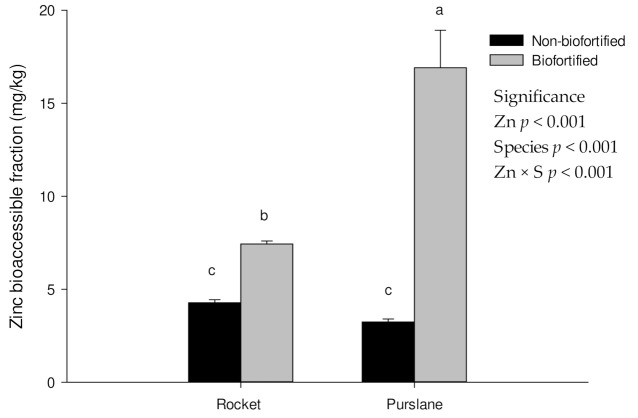
Bioaccessible fraction (mg/kg) of Zn in non-biofortified and biofortified rocket and purslane after in vitro digestion process. Results are reported as mean ± standard error of treatment (*n* = 3). Different letters indicate that mean values are significantly different (means separation by LSD; α = 0.05). Non-biofortified (0.13 mg/L of Zn in nutrient solution), Biofortified (5.2 mg/L of Zn in nutrient solution).

The BF is the concentration of a nutrient or a bioactive compound (mineral or organic) that is extracted from the plant matrix during the digestion process and which, potentially, becomes bioavailable in the intestinal tract. The number of mineral elements released by plant materials is related to various factors such as species, food processing (raw or cooked food), texture, nutrient concentration, and interaction with other nutrients or antinutrients [17,32,42,45,46]. In our study, after in vitro gastrointestinal digestion, Zn BF_%_ was 98% in biofortified plants and 73% in non-biofortified plants compared to the non-digested control plants. Similar results were reported for Si-biofortified green bean pods [17]. Conversely, no differences in BF_%_ values (72%) were found in rocket (biofortified and non-biofortified), although an increase in Zn was found in the edible parts (Figure 1). Therefore, the in vitro digestion protocol allows similar BF_%_ values to be obtained in both biofortified and non-biofortified plants. This result was also reported in our previous study [32,47], showing that increasing the concentration of mineral elements in the edible parts of biofortified plants does not always give an increase in BF_%_, as reported for calcium and silicon [32,47]. However, in both rocket and purslane, after the in vitro gastrointestinal digestion (bioaccessible fraction), we measured a significant release of Zn (mg/kg) in biofortified plants compared to non-biofortified ones (76% and 298%, respectively, for rocket and purslane), as shown in Figure 2. Biofortified purslane was found to be the species with the highest amount of bioaccessible Zn released during the digestion process (16.9 mg/kg). The quantity of Zn released by biofortified rocket was 7.43 mg/kg. The quantity of bioaccessible Zn released by non-biofortified purslane and rocket was 3.75 mg/kg (on average).

As previously reported also in soybean sprouts [42], the BF of Zn, measured after in vitro gastrointestinal digestion, is affected by the initial content of Zn in the edible parts of the plants. The increase in the amount of Zn released during the digestion process and found in this study is a significant result, considering that this is the amount of Zn that could be potentially absorbed in the intestinal tract [48].

The BF of the mineral elements is correlated to the different compositions of the tested species and to the interaction of the plants with the intestinal juices (pancreatic enzymes and bile salts). As reported in Table 4, all mineral elements analyzed showed significant differences (*p* < 0.001) in relation to the plant species, but they were not affected by the Zn biofortification protocol used. The influence of the plant species on BF values has also been found in other studies analyzing various mineral elements, such as Si [47], Ca [32,49], K [45,49], Fe [6], Mg [49], and other trace elements [49]. In our study, the average quantities of mineral elements released in the digestion process were 0.53 mg/kg for Al, 2.36 mg/kg for B, and 7522 mg/kg for K, and these quantities were higher in rocket than in purslane. Conversely, the measured mean amounts of Fe (2.12 mg/kg) and Mg (880 mg/kg) were higher in purslane than in rocket (Table 4).

Several compounds, such as some antinutritional factors (carbonate, phytic and oxalic acids) and some healthy food components (proteins, fibers, and polyphenols), can modify the release of nutrients from the food matrix [50]. The interaction of mineral elements with these compounds generates insoluble salts and determines the reduction of BF and a reduced absorption of minerals [30,31]. Egea-Gilabert et al. [22] reported that purslane is a vegetable with a high oxalate content (2000 mg/kg of fresh weight). On the contrary, rocket is generally considered to be free of oxalate [23]. This difference in oxalate content could influence the BF of all mineral elements evaluated: in particular, Ca and Sr. Oxalate forms an insoluble salt with Ca [51] and probably also with Sr, considering the similar chemical and biological properties of these mineral elements [52]. The effects of plant species on Ca bioaccessibility and the high amount of Ca released during the digestion process were reported in our previous study [32].

The highest amounts of Ca and Sr in the digested liquid were found in the non-biofortified rocket, followed by the biofortified rocket, whereas the purslane released lower amounts of Ca in the gastrointestinal digestion, and this result was not affected by the biofortification treatment with Zn. The high amounts of Ca observed in rocket could lead to the formation of low-solubility complexes that reduce the BF of Mn. Furthermore, mineral elements with similar electronic configurations (Zn^2+^, Ca^2+^, Fe^2+^, Mg^2+^, Mn^2+^, and Sr^2+^) are involved in mechanisms of mutual competition to bind antinutrient compounds [27,28,46]. Therefore, different values of BF and BF_%_ can be attributable to different factors, including the mechanisms of competition at different levels in a plant-based food system.

### 3.4. Daily Intake, Coverage of RDA-Zn (Male and Female), and Hazard Quotient

The DI, the RDA-Zn coverage (for men and women), and the HQ for Zn intake through digesting 100 g of baby leaf vegetables (average servings for this type of products) are shown in Table 5. The Zn biofortification significantly increased those parameters (*p* < 0.001), and differences between the two vegetables were found (Table 5). The highest values of DI, RDA-Zn coverage, and HQ were obtained for biofortified purslane, whereas the lowest values were found for non-biofortified rocket and purslane (Table 5). After digestion of 100 g of biofortified purslane, an increase in DI (3.9-fold) and RDA-Zn coverage was found in males and females, compared to non-biofortified vegetables (Table 5).

The increase of DI and RDA-Zn coverage accentuates the efficiency of the applied biofortification protocol, suggesting its use to produce Zn-biofortified baby leaf vegetables for different target consumers groups for which the increase of the DI is advisable, such as pregnant and breastfeeding women, vegetarians/vegans, people with various diseases, and the elderly [7].

The HQ values found in rocket (biofortified and not) and in non-biofortified purslane were less than 1. However, an excessive increase of Zn in the edible portions of purslane can result in an increase of this parameter. When the HQ is higher than 1, adverse health effects are likely to occur. According to our findings, the consumption of 100 g of our biofortified products does not pose any health risk to consumers. This aspect must be taken into due consideration when approaching a biofortification process; an excessive content of Zn in the edible parts of vegetables would represent a risk for consumers (the maximum tolerable intake level is 40 mg Zn/day) since vegetables are only a relative portion of the diet and other foods and water intake can significantly contribute to the daily intake of Zn [39].

## 4. Conclusions

The general purpose of this study was to produce Zn-biofortified rocket and purslane and to propose a workflow for studying their nutritional qualities based on the analysis of the bioaccessible fraction of the overall mineral elements.

The agronomic biofortification protocol used in this study was based on increasing the concentration of Zn in the NS used for the cultivation of rocket and purslane in soilless conditions. This protocol allowed Zn-biofortified plants with a higher nutritional quality to be obtained. The amount of bioaccessible Zn released by the plants during the digestion process was influenced by the species (rocket and purslane) and by the initial Zn content accumulated in the edible parts of the plants in soilless cultivation using Zn-enriched NS.

The use of the in vitro gastrointestinal digestion protocol allowed the evaluation of the bioaccessible fraction of Zn and other mineral elements. Antinutritional factors (carbonate, phytic and oxalic acids) and some healthy food components (proteins, fibers, and polyphenols) can modify the release of nutrients from the food matrix, generating insoluble salts and determining the reduction of bioaccessibility and absorption of the mineral elements. Hence, it is important to quantify the bioaccessible fraction of the target mineral and also of the other mineral elements.

Our results confirmed that in vitro digestion is a valuable method for assessing the nutritional efficiency of the biofortification process. This approach can be efficiently used to improve the design process for biofortified products. Furthermore, the calculated hazard quotient demonstrates the safety of biofortified rocket and purslane.

Overall, the consumption of biofortified rocket and purslane would provide greater intake of Zn in the human diet without causing harm to the consumer, thus, providing benefits for different classes of consumers, such as the elderly, vegetarians, vegans, and people with gastrointestinal and other diseases. However, more research is needed to further explore and validate the applicability of the proposed workflow to biofortification processes for other mineral elements and in other plant species.

## Figures and Tables

**Table 1:** Mineral content recovered from certified reference materials (NIST tomato leaf 1535a), LOD, and LOQ of methods.

Element	LOD	LOQ	Found	Certified	Recovery
	µg/L		mg/kg DW		(%)
Al	0.8904	2.6982	624 ± 33.65	598 ± 7.1	104
B	0.0451	0.1365	29.7 ± 0.29	33.0 ± 0.42	90
Ca	0.0698	0.2116	49,437 ± 113.4	50,450 ± 550	98
Fe	0.2923	0.8853	358.3 ± 0.92	367 ± 4.3	98
K	0.7344	2.2255	30,443 ± 99	26,760 ± 480	113
Mg	0.1458	0.4420	11,649 ± 35.03	12,000	97
Mn	0.1898	0.5752	264.1 ± 1.24	246 ± 7.1	107
Sr	0.2068	0.6267	88.0 ± 0.401	85.0	104
Zn	0.1763	0.5343	30.7 ± 0.205	30.9 ± 0.55	99

Results are reported as mean ± standard error. Magnesium and strontium: non-certified value. Insufficient information is available to assess the uncertainty associated with the value, and, therefore, no uncertainty is provided (NIST).

**Table 2:** Mineral content recovered from bioaccessibility assays of certified reference materials (NIST tomato leaf 1535a).

Element	BF	Residue	MB	Certified	BF_%_	Recovery
	mg/kg				(%)	
Al	25.0 ± 0.335	309 ± 18.78	334 ± 18.45	598 ± 7.1	4.19 ± 0.05	56 ± 3.08
B	21.8 ± 0.833	10.1 ± 0.099	32 ± 0.82	33 ± 0.42	66.1 ± 2.52	97 ± 2.49
Ca	31411 ± 149	16,310 ± 718	47,720 ± 867	50,450 ± 550	62.3 ± 0.29	95 ± 1.71
Fe	18.6 ± 0.03	228 ± 11.98	247 ± 12.03	367 ± 4.3	5.1 ± 0.009	67 ± 3.27
K	19,653 ± 161	589 ± 19.38	20,242 ± 182	26,760 ± 480	73.4 ± 0.61	76 ± 0.79
Mg	12,807 ± 82.3	545 ± 30.58	13,351 ± 113	12,000	107 ± 0.69	111 ± 0.94
Mn	213.6 ± 0.156	50.2 ± 2.103	264 ± 2.259	246 ± 7.1	86.8 ± 0.06	107 ± 0.92
Sr	58.9 ± 0.668	30.8 ± 1.408	90 ± 2.147	85.0	69.2 ± 0.79	105 ± 2.52
Zn	18.9 ± 0.664	10.2 ± 0.66	29 ± 1.332	30.9 ± 0.55	61.2 ± 2.15	94 ± 4.31

Results are reported as mean ± standard error. Magnesium and strontium: non-certified value. Information available is not sufficient to assess the uncertainty associated with the value, and, therefore, no uncertainty is provided (NIST). BF: bioaccessible fraction = concentration of element release from plant material during in vitro digestion process. Residue: residual concentration of the element in digested samples. MB: mass balance = BF + Residue. Certified: the certified value from the National Institute of Standards and Technology (NIST). BF_%_: bioaccessibility = (BF/Certified) × 100. Recovery = (MB/certified) × 100.

**Table 3:** Mineral content in non-biofortified and biofortified rocket and purslane harvested at the phenological stage of “baby leaf vegetables”.

		Al	B	Ca	Fe	K	Mg	Mn	Sr
Species	Treatment	mg/kg of Fresh Weight							
Rocket	Non-biofortified	3.27 ± 0.13	2.66 ± 0.05	3155 ± 288	6.56 ± 0.28	7084 ± 545	482 ± 36.7	2.02 ± 0.02	6.23 ± 0.12
	Biofortified	3.37 ± 0.25	2.72 ± 0.09	3572 ± 80.9	6.81 ± 0.15	7657 ± 627	556 ± 20.9	2.46 ± 0.16	6.10 ± 0.36
	Significance	ns	ns	ns	ns	ns	ns	ns	ns
Purslane	Non-biofortified	0.89 ± 0.13	2.32 ± 0.06	875 ± 26.5	4.21 ± 0.17	4373 ± 199	818 ± 18.3	7.66 ± 0.34	2.54 ± 0.07
	Biofortified	0.89 ± 0.07	2.41 ± 0.23	1004 ± 67.8	3.93 ± 0.31	4184 ± 212	894 ± 55.4	9.44 ± 0.91	3.00 ± 0.155
	Significance	ns	ns	ns	ns	ns	ns	ns	ns

Results are reported as mean ± standard error of treatment (*n* = 3). Significance: ns = not significant. Means separation within columns by LSD (α = 0.05). Non-biofortified (0.13 mg/L of Zn in nutrient solution), Biofortified (5.2 mg/L of Zn in nutrient solution).

**Table 4:** Bioaccessible fractions of Al, B, Ca, Fe, K, Mg, Mn, and Sr in non-biofortified and biofortified rocket and purslane after in vitro digestion process.

		Al	B	Ca	Fe	K	Mg	Mn	Sr
Species	Treatment	mg/kg of Fresh Weight							
Rocket	Non-biofortified	0.57 ± 0.02	2.28 ± 0.22	2352 ± 50.0 ^a^	1.24 ± 0.12	7428 ± 342	437 ± 13.4	1.56 ± 0.05 ^c^	6.15 ± 0.16 ^a^
	Biofortified	0.49 ± 0.05	2.44 ± 0.22	2232 ± 1.47 ^b^	1.4 ± 0.07	7617 ± 254	466 ± 2.99	1.61 ± 0.06 ^c^	5.57 ± 0.04 ^b^
Purslane	Non-biofortified	0.09 ± 0.01	1.77 ± 0.08	59.6 ± 2.39 ^c^	2.33 ± 0.08	4422 ± 75.2	818 ± 25.1	7.33 ± 0.40 ^b^	1.14 ± 0.02 ^c^
	Biofortified	0.09 ± 0.01	1.96 ± 0.06	63.8 ± 3.96 ^c^	1.91 ± 0.27	4104 ± 41.8	880 ± 83.5	8.53 ± 0.29 ^a^	1.29 ± 0.06 ^c^
Significance									
Zn		ns	ns	ns	ns	ns	ns	ns	ns
Species (S)		***	**	***	***	***	***	***	***
Zn × S		ns	ns	*	ns	ns	ns	*	**

Results are reported as mean ± standard error of treatment (*n* = 3). FW: fresh weight. Significance: ns = not significant; * *p* ≤ 0.05; ** *p* < 0.01; *** *p* ≤ 0.001. Different letters within column indicate that mean values are significantly different (means separation by LSD; α = 0.05). Non-biofortified (0.13 mg/L of Zn in nutrient solution), Biofortified (5.2 mg/L of Zn in nutrient solution).

**Table 5:** Daily intake, coverage of RDA for Zn, and HQ for Zn intake through consumption of 100 g portions of baby leaf vegetables, biofortified and non-biofortified, by adult male and female humans (70 kg body weight).

		Daily Zn Intake (mg Zn/Day)	RDA-Zn Coverage (%)		HQ
Species	Treatment		Male	Female	
Rocket	Non-biofortified	0.43 ± 0.02 ^c^	3.88 ± 0.14 ^c^	5.34 ± 0.21 ^c^	0.278 ± 0.011 ^c^
	Biofortified	0.74 ± 0.02 ^b^	6.79 ± 0.14 ^b^	9.29 ± 0.20 ^b^	0.534 ± 0.012 ^b^
Purslane	Non-biofortified	0.32 ± 0.02 ^c^	2.94 ± 0.15 ^c^	4.05 ± 0.20 ^c^	0.233 ± 0.017 ^c^
	Biofortified	1.69 ± 0.19 ^a^	15.4 ± 1.83 ^a^	21.14 ± 2.52 ^a^	1.086 ± 0.129 ^a^
Significance					
Zn		***	***	***	***
Species (S)		***	***	***	***
Zn × S		***	***	***	***

Results are reported as mean ± standard error of treatment (*n* = 3). Significance: *** *p* ≤ 0.001. Different letters within columns indicate that mean values are significantly different (means separation by LSD; α = 0.05). Daily intake, coverage of RDA for Zn, and HQ were calculated in relation to the quantity of Zn released from vegetables during the gastrointestinal digestion process. Major details are reported in Section 2.5 of Materials and Methods.
